# Internet-Based Cognitive Therapy for Social Anxiety Disorder in Hong Kong: Therapist Training and Dissemination Case Series

**DOI:** 10.2196/13446

**Published:** 2019-05-15

**Authors:** Graham R Thew, Candice LYM Powell, Amy PL Kwok, Mandy H Lissillour Chan, Jennifer Wild, Emma Warnock-Parkes, Patrick WL Leung, David M Clark

**Affiliations:** 1 Department of Experimental Psychology University of Oxford Oxford United Kingdom; 2 Oxford University Hospitals National Health Service Foundation Trust Oxford United Kingdom; 3 Oxford Health National Health Service Foundation Trust Oxford United Kingdom; 4 New Life Psychiatric Rehabilitation Association Hong Kong China (Hong Kong); 5 New Territories East Cluster Hospital Authority Hong Kong China (Hong Kong); 6 Kowloon Central Cluster Hospital Authority Hong Kong China (Hong Kong); 7 Kings College London London United Kingdom; 8 The Chinese University of Hong Kong Hong Kong China (Hong Kong)

**Keywords:** anxiety, social phobia, internet, cognitive therapy, clinical competence, cross-cultural comparison, Hong Kong, benchmarking, psychology, clinical, mental health

## Abstract

**Background:**

Guided internet-based psychological interventions show substantial promise for expanding access to evidence-based mental health care. However, this can only be achieved if results of tightly controlled studies from the treatment developers can also be achieved in other independent settings. This dissemination depends critically on developing efficient and effective ways to train professionals to deliver these interventions. Unfortunately, descriptions of therapist training and its evaluation are often limited or absent within dissemination studies.

**Objective:**

This study aimed to describe and evaluate a program of therapist training to deliver internet-based Cognitive Therapy for social anxiety disorder (iCT-SAD). As this treatment was developed in the United Kingdom and this study was conducted in Hong Kong with local therapists, an additional objective was to examine the feasibility, acceptability, and initial efficacy of iCT-SAD in this cultural context, based on data from a pilot case series.

**Methods:**

Training in iCT-SAD was provided to 3 therapists and included practice of the face-to-face format of therapy under clinical supervision, training workshops, and treating 6 patients with the iCT-SAD program. Training progress was evaluated using standardized and self-report measures and by reviewing patient outcomes. In addition, feedback from patients and therapists was sought regarding the feasibility and acceptability of the program.

**Results:**

The training program was effective at increasing therapists’ iCT-SAD knowledge and skills, resulting in levels of competence expected of a specialist Cognitive Behavioral Therapy practitioner. The 6 patients treated by the trainees all completed their treatment and achieved a mean pre- to posttreatment change of 53.8 points (SD 39.5) on the primary patient outcome measure, the Liebowitz Social Anxiety Scale. The within-group effect size (Cohen *d*) was 2.06 (95% CI 0.66-3.46). There was evidence to suggest that the patients’ clinical outcomes were sustained at 3-month follow-up. These clinical results are comparable to those achieved by UK patients treated by the developers of the internet program. Patient and therapist feedback did not identify any major cultural barriers to implementing iCT-SAD in Hong Kong; some modest language suggestions were made to assist understanding.

**Conclusions:**

The therapist training implemented here facilitated the successful dissemination of an effective UK-developed internet intervention to Hong Kong. The treatment appeared feasible and acceptable in this setting and showed highly promising initial efficacy. A randomized controlled trial is now required to examine this more robustly. As therapist training is critical to the successful dissemination of internet interventions, further research to develop, describe, and evaluate therapist training procedures is recommended.

## Introduction

### Background

The delivery of psychological therapies via the internet has received a good deal of research attention in recent years. Multiple randomized controlled trials (RCTs) have shown that CBT-based internet interventions can be effective in treating depression and anxiety disorders when compared with no treatment [1-3], with the effects being larger when patients’ online work is guided and supported by a clinician or coach [4-6]. Some guided internet treatments have also shown results comparable to face-to-face treatment in RCTs, despite requiring much less clinician time [1,7-9]. These findings suggest that internet therapies have substantial potential for expanding access to effective psychological treatments, which is critical given the undertreatment of common mental health problems such as social anxiety in almost all countries [10]. However, this potential will only be realized if the results that are obtained by the developers of these internet interventions in tightly controlled RCTs can be maintained when those interventions are made available in other settings, providing treatment routinely and with guidance from clinicians who were not involved in developing the program. Whether this is possible will depend critically on the extent to which the field can develop efficient and effective ways of training clinicians to support and guide patients going through these interventions. Large-scale dissemination of therapist-guided interventions will only be achievable if effective therapist training methods are in place.

Promisingly, several studies have attempted to disseminate internet therapies beyond the teams that have developed the interventions and have reported promising results. These include the translation and trialing of Swedish internet-based Cognitive Behavioral Therapy interventions in Romania [11] and Norway [12], a Swiss intervention implemented in China [13], and a Spanish intervention trialed in the Netherlands [14]. However, none of these studies have provided sufficient detail about how the therapists or coaches in the dissemination sites were trained and how the effectiveness of the training was evaluated. There is a clear need for such descriptions if the field is to reliably succeed with dissemination. This study aimed to address this by describing in detail an example of how clinicians in Hong Kong were trained to deliver an effective internet therapy for social anxiety disorder (SAD) that was developed in the United Kingdom and how this training was evaluated.

The internet-based Cognitive Therapy for social anxiety disorder (iCT-SAD) program is based on Clark and Wells’ face-to-face Cognitive Therapy for SAD (CT-SAD). CT-SAD has strong empirical support for the treatment of SAD, showing efficacy superior to that of various alternative treatments in RCTs [15-18] and a network meta-analysis [19]. As a result, the UK National Institute for Health and Care Excellence recommends CT-SAD as a first-line treatment for adults with SAD [20]. The iCT-SAD program implements all the procedures of face-to-face CT-SAD online, including special features such as video feedback of social performance and behavioral experiments that allow patients to test out their feared concerns. It includes multiple brief video clips illustrating key assignments that patients are encouraged to do to challenge their fearful beliefs, as modeling has been shown to be one of the most effective ways of reducing anxiety [21]. The treatment is delivered through a series of online modules, which include core modules given to all patients, and a range of optional modules targeting different specific concerns (eg, blushing, feeling boring, worrying in advance). These allow the therapist to tailor the treatment to patients’ individual needs. As most of the therapy content is delivered by the program, iCT-SAD greatly reduces the amount of therapist time required per patient compared with face-to-face CT-SAD [22]. The aim of iCT-SAD is that most of the key learning that occurs in the treatment can be achieved by the patient’s self-study on the internet, facilitated by therapists who are themselves skilled in delivering the face-to-face treatment. This online facilitation draws strongly on the clinical knowledge expected of an experienced clinician but asks them to apply this in a very different way. The program introduces the key concepts that would normally be introduced by the therapist and guides much of the patient’s learning. Therefore, the therapist must carefully monitor patients’ progress within the program, looking closely at what the patient has and has not already learnt, and then make clinically informed suggestions to help them deepen and extend their learning. For example, they may support the patient to plan new behavioral experiments or direct them to other sections of the program that are most appropriate for their particular concerns. Therefore, it may be particularly critical to pay close attention to how therapists are trained to deliver an internet intervention of this sort, given that the therapist’s role, and the application of their clinical knowledge, is different compared with face-to-face interventions.

As this study involved dissemination to a different culture as well as to a different team, it also had to consider issues about the extent of cultural adaptation of the treatment that would be required. One approach to this problem would be to assume that if the treatment is delivered in a different culture it has to be adapted in ways beyond linguistic translation alone and that this must be done before any evaluation of the treatment in the new context. An alternative approach is to first pilot the intervention in its unadapted form to benchmark it against results from studies in its country of origin and, at the same time, to obtain feedback from clients and therapists about what adaptations they think might be needed. This latter approach, which aims to identify the minimum number of adaptations required, was taken for this study.

### Objectives

This study therefore aimed to describe and evaluate a program of therapist training for iCT-SAD. As this treatment was developed in the United Kingdom and this study was conducted in Hong Kong with local therapists, an additional objective was to examine the feasibility, acceptability, and initial efficacy of iCT-SAD in this cultural context, based on data from a pilot case series. We aimed to identify whether significant cultural adaptations would be required for further dissemination in this setting.

## Methods

### Therapist Training Procedure

The training consisted of 3 phases, outlined below:

#### Phase 1: Initial Face-To-Face Cases Under Supervision

As iCT-SAD is a direct adaptation of the face-to-face Cognitive Therapy (CT) protocol, it is important that therapists are familiar with CT before learning internet-based Cognitive Therapy (iCT). This permits a greater understanding of the key points the online modules are aiming to convey.

Training for this study therefore started with 3 therapists from Hong Kong undertaking a phase of face-to-face CT-SAD. The therapists had been qualified as clinical psychologists for an average of 10.3 years (7, 9, and 15 years). All spoke English as an additional language. Although they were experienced in delivering cognitive-behavioral interventions for anxiety, including SAD, and 2 of them had previously attended a CT-SAD workshop, none of them had used the full CT protocol previously. The therapists were sent the most recent version of the treatment manual [23], which explains the CT-SAD protocol as applied to adults as well as how treatment is adapted for adolescents. The 3 therapists then implemented the treatment in Cantonese with a total of 5 cases between January and May 2017, with weekly group supervision from the first author via Skype, which focused on learning and implementing the treatment protocol.

#### Phase 2: Internet-Based Cognitive Therapy for Social Anxiety Disorder Training

The second phase consisted of training in the iCT-SAD program and treatment protocol. The iCT-SAD training was developed and delivered by the authors (GT, DMC, and JW) and was informed by the results of interviews with 3 iCT-SAD therapists from the United Kingdom who were involved in the development of the treatment as well as the first author’s own experience of learning and delivering iCT-SAD. Training was delivered in an intensive format, totaling 7.5 days over a 2-week period. Each therapist was registered with an account on the iCT-SAD site, together with a *test patient* account to allow them to view the site content from the patient’s perspective and to practice using the site within the training sessions. The training was divided into the following sections:

##### Site Navigation and Functionality

Learning to navigate the site and use the site functions, such as releasing treatment modules, sending messages, and using the resource library.

##### Structure and Timings

Learning how the iCT-SAD treatment protocol is implemented over the treatment period, for example, when each core module is released and when to schedule phone calls.

##### Module Content

The majority of the training focused on reviewing the content and general aims of each module to help the therapists know how to review and discuss modules with patients.

##### Therapist Communication

Effective use of the iCT-SAD communication methods (messaging, short message service (SMS), and phone calls) was demonstrated and practiced by the therapists using role play.

##### Implementing Key Techniques

As some of the key therapy techniques from CT, such as developing an individualized model, and the *self-focused attention and safety behaviors experiment* are implemented slightly differently online, time was allocated to review and practice these specifically.

##### Behavioral Experiments

The training included experiential practice of planning and completing behavioral experiments in real social situations. The aim was to support the therapists to generate ideas for appropriate experiments and to provide them with first-hand experience to draw on when discussing experiments in iCT-SAD.

##### The Role of the Therapist in Internet-Based Cognitive Therapy for Social Anxiety Disorder

Training focused on understanding the role of the therapist in iCT-SAD as a guide and facilitator, how they apply their clinical expertise, and how this differs from face-to-face work.

##### Troubleshooting

Common problems, such as patients not logging into the site, low motivation, or avoidance of behavioral experiments, were discussed and possible solutions reviewed.

#### Phase 3: Internet-Based Cognitive Therapy for Social Anxiety Disorder Pilot Cases Under Supervision

Phase 3 consisted of piloting the iCT-SAD treatment with a small number of cases under weekly group clinical supervision from the first author via Skype. Each therapist treated 2 clients from a local clinical service in Hong Kong using the program. Supervision focused on reinforcing learning from Phase 2, adherence to the treatment protocol, and further development of skills. The therapist training and a subsequent clinical trial were approved by the Joint Chinese University of Hong Kong—New Territories East Cluster Clinical Research Ethics Committee (Ref: 2016.611-T) and by the University of Oxford Tropical Research Ethics Committee (Ref: 531-17).

### Training Evaluation

The training was evaluated using the following 4 methods:

#### Face-to-Face Cognitive Therapy for Social Anxiety Disorder: Cognitive Therapy Competence Scale for Social Phobia

The Cognitive Therapy Competence Scale for Social Phobia (CTCS-SP) [24] (also available in an unpublished manuscript by Clark et al) is a disorder-specific adaptation of the general Cognitive Therapy Scale—Revised (CTS-R) [25], allowing the assessor to rate skills and techniques specific to the face-to-face CT-SAD treatment protocol. This competence measure has been shown to be a strong predictor of patient outcomes [26]. The CTCS-SP contains 15 skill areas, each rated on a 0 to 6 scale, with higher scores indicating greater competence. A mean score of 3 (ie, 50%) is considered the threshold to demonstrate competence in delivering the treatment. During Phase 1 (face-to-face CT training), 1 complete session from each therapist was rated by the first author using a video of the session (in Cantonese) together with an English transcript of the conversation. Sessions were chosen by each therapist, but could not include the first or last sessions, and were required to include at least one behavioral experiment. Feedback from these assessments was provided in a subsequent supervision session.

#### Internet-Based Cognitive Therapy for Social Anxiety Disorder Self-Evaluation Assessment

This self-report assessment was developed specifically for this study. It asks respondents to rate their own knowledge and skills in relation to 34 internet therapy components and activities, including those applicable to most online interventions (eg, “Developing and maintaining client engagement with the online programme”) and those more specific to iCT-SAD (eg, “Suggesting strategies to overcome problems clients may experience with video feedback”). An initial item pool was generated through interviews with 3 experienced UK iCT-SAD trial therapists and their supervisor, which was then reviewed and refined in consultation with these experts to ensure face validity. For each item, respondents provide 1 rating for knowledge (ie, how much they know about the item) and 1 for skills (ie, how well they think they can currently implement it). Ratings are given on 0 to 8 Likert scales, where 0, 2, 4, 6, and 8 represent *no*, *limited*, *some*, *good*, and *extensive* knowledge or skills, respectively. Mean scores for each subscale are calculated. The full assessment is provided in Multimedia Appendix 1. The therapists completed the assessment following each phase of training.

#### Internet-Based Cognitive Therapy for Social Anxiety Disorder Skills Test

To obtain a more objective assessment of what the therapists had learned following Phase 2, a standardized skills test was developed and completed by the therapists at this timepoint. The test consisted of brief information about a series of fictional patients undertaking iCT-SAD. For each, an extract of some completed work on the site is shown and the respondent is asked questions about what the patient has written and how they would proceed with the treatment. For example, questions might ask how they would help a patient to improve their individualized cognitive model or to write a response to a message where the patient reports a drop in mood (The test is provided in Multimedia Appendix 2). Items were generated following the same iterative process of consultation with iCT-SAD experts as used for the self-evaluation assessment. Respondents’ answers are scored in relation to set of exemplar responses generated by iCT-SAD experts. Exemplar responses were divided into distinct components, with a point awarded for each one mentioned by the respondent. The maximum score was 55.

#### Internet-Based Cognitive Therapy for Social Anxiety Disorder Patient Outcomes

The final method to evaluate the training effectiveness was to examine the outcomes of the pilot patient cohort being treated by the therapists using the program. This study also provided an initial test of the iCT-SAD program in Hong Kong, examining its feasibility, acceptability, and technical functioning, while also offering a tentative indication of efficacy and cultural applicability.

### Internet-Based Cognitive Therapy for Social Anxiety Disorder Pilot Case Series

#### Participants

A cohort of 6 patients (3 female; mean age 31.3 years, age range 18 to 49 years) was recruited from the New Life Psychiatric Rehabilitation Association, Hong Kong. The cohort represented a consecutive series of referrals for probable social anxiety. All the patients met the Diagnostic and Statistical Manual 4^th^ Edition (DSM-IV) criteria for SAD, which was confirmed at assessment using the Anxiety Disorders Interview Schedule for DSM-IV [27]. Within the cohort, 4 patients showed generalized social anxiety, whereas 2 experienced anxiety in a more restricted range of situations. The mean duration of social anxiety was 7.5 years (SD 8.8). Comorbidities were assessed using the Structured Clinical Interview for DSM-5 [28]. SAD was the primary diagnosis for all 6 patients, but 1 met the criteria for comorbid Generalized Anxiety Disorder (GAD), 1 for comorbid major depressive disorder, and 1 for comorbid GAD, other specified depressive disorder, panic disorder, agoraphobia, specific phobia, and separation anxiety. The remaining 3 only met the criteria for SAD. Assessments were conducted by a trainee clinical psychologist under supervision. All patients were Chinese residents of Hong Kong, with non-native but sufficient proficiency in English to undertake the treatment in its original language. All participants were in full-time employment or study. Regarding marital status, 2 participants were married or cohabiting and 4 were single.

#### Procedure

The iCT-SAD program, which was written in English, was implemented as described by Stott et al [22]. Therapists communicated with their patients using brief secure messages, telephone calls, and occasional webcam chats. Therapists’ messages were written in English, and the telephone calls were conducted in both English and Cantonese as required. The treatment lasted 14 weeks, followed by a 3-month booster period, during which participants retained access to the program. At the end of treatment, participants were invited to complete a brief online survey about their experience of treatment and their suggestions for how it could be improved. Questions examined the ease of understanding the treatment in English, helpfulness of therapists’ behaviors, overall likes and dislikes, and general ease of use. The respondents were not asked to give their name and completed the survey outside of the treatment website, meaning the therapists could not see their responses. The therapists were also invited to share their feedback and suggestions during a focus group discussion within clinical supervision. This centered on the cultural applicability of the treatment and the need for any adaptation in future.

#### Measures

The primary patient outcome measure was the self-report version of the Liebowitz Social Anxiety Scale (LSAS) [29]. Secondary patient outcome measures included other measures of SAD to facilitate comparison with other studies, process measures, and assessments of general mood and functioning:

Social Anxiety:

- Social Phobia Weekly Summary Scale [30]
- Social Phobia Inventory (SPIN) [30,31]
- Fear of Negative Evaluation Scale [30,32].
- Social Phobia Scale (SPS) and Social Interaction Anxiety Scale [30,33].

Social anxiety process measures:

- Social Cognitions Questionnaire [30]
- Social Behavior Questionnaire (SBQ) [30]
- Social Attitudes Questionnaire (SAQ) [30]
- Social Participation and Social Satisfaction scales [34]

General Mood and Functioning Measures:

- Patient Health Questionnaire (PHQ)—9-item version [35]
- Generalized Anxiety Disorder Questionnaire (GAD)—7-item version [36]
- Work and Social Adjustment Scale [37]

#### Analysis of Clinical Data

Mean scores on each measure at pretreatment, posttreatment, and 3-month follow-up were examined. Given the small sample size, these were evaluated using effect size estimates and 95% CIs rather than significance tests. In addition, the reliability of each patient’s change in scores was examined using the response and remission criteria outlined below. Effect sizes (Cohen *d*) were calculated using the pooled SD as the denominator, calculated as SQRT((SD^2^_initial_+SD^2^_post_)/2) [38]. Interpretation followed the rules of thumb outlined by Cohen [39], with effect sizes of 0.2, 0.5, and 0.8 indicating small, medium, and large effects, respectively. CIs for Cohen *d* were calculated using the Hedges and Olkin formula [40].

#### Response and Remission Criteria

For classifying patients as treatment responders and/or remitted social anxiety, we used the criteria described by Stott et al’s [22] original iCT-SAD case series, which use both the reliable change formulae described by Jacobson and Truax [41] and normative data from Fresco et al [42]. Response to treatment was defined as an improvement greater than 31% on the LSAS between pretreatment and posttreatment [43]. Remission was defined as a drop of at least 12 points on the LSAS between pretreatment and posttreatment combined with a posttreatment score at or below the clinical threshold of 38 points.

We also examined reliable improvement and reliable recovery from social anxiety using the English *Improving Access to Psychological Therapies* (IAPT) [44] outcome criteria, which consider change on both anxiety disorder–specific (SPIN) and depressed mood (PHQ) measures [45]. Reliable improvement was defined as a decrease of 10 or more points on the SPIN and/or 6 or more points on the PHQ and no reliable deterioration (increases of 10 or more, or 6 or more, respectively) on either measure. Reliable recovery was defined as reliable improvement combined with scores on both measures below the clinical threshold, that is, a SPIN score of 18 or below and a PHQ score of 9 or below.

## Results

### Face-to-Face Cognitive Therapy for Social Anxiety Disorder: Cognitive Therapy Competence Scale for Social Phobia

Mean CTCS-SP scores across the 15 face-to-face CT skill areas were 3.9, 3.9, and 4.0 across the 3 therapists. These scores indicated that all 3 therapists achieved a level of competence in the delivery of the face-to-face treatment that would be expected of a high-intensity IAPT therapist (mean score >3).

### Internet-Based Cognitive Therapy for Social Anxiety Disorder Self-Evaluation Assessment

**Figure 1:**
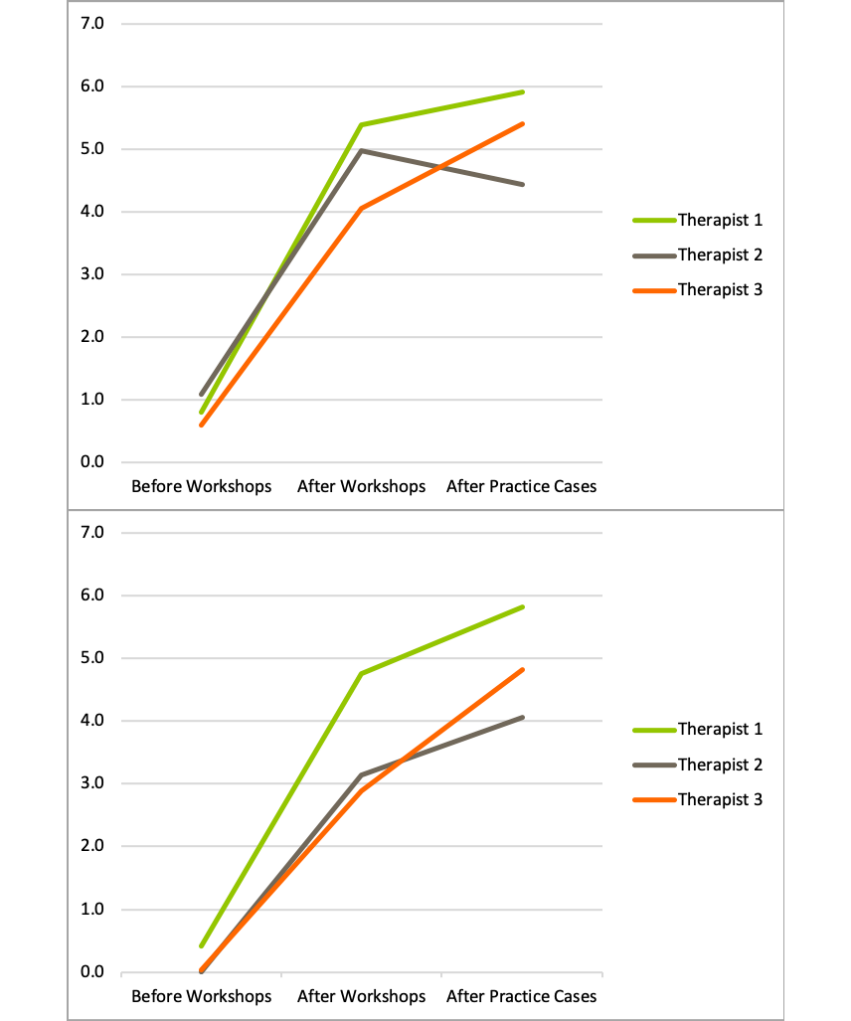
Therapists’ self-reported knowledge (top panel) and skills (bottom panel) ratings for internet-based Cognitive Therapy for social anxiety disorder following each phase of training.

Therapists’ self-reported knowledge and skills ratings across the 3 timepoints are shown in Figure 1. This figure shows that at baseline, where therapists had been trained in the face-to-face CT protocol but not iCT, self-reported iCT knowledge and skills ratings were low. These mean scores increased following the iCT training workshops in Phase 2 and further increased following completion of iCT practice cases under supervision in Phase 3.

### Internet-Based Cognitive Therapy for Social Anxiety Disorder Skills Test

The 3 therapists’ scores on the different components of the skills test are shown in Table 1. As this is a newly developed instrument, there are no established competency cutoffs. However, as the test was designed in a similar manner to the CTCS-SP, with each item examining competence in a distinct skill, the CTCS-SP competency threshold of 50% is thought to be broadly applicable to the skills test and is used here to aid interpretation.

**Table 1:** Therapists’ scores on each component of the skills test.

Skill assessed	Maximum score	Therapist 1	Therapist 2	Therapist 3
Interpreting client questionnaire responses and treatment planning	16	12	13	11
Reviewing patients’ individualized cognitive models	9	6	4	4
Conducting the *self-focused attention and safety behaviors experiment*	3	3	2	3
Planning phone calls	5	3	4	4
Reviewing patients’ behavioral experiments	6	5	5	6
Reviewing patients’ writing in modules	5	3	1	2
Responding to a patient message	7	4	2	3
Reviewing patients’ therapy blueprints	4	3	2	2
Total	55	39 (71%)	33 (60%)	35 (64%)

In this skills test, the 3 therapists achieved overall scores of 71%, 60%, and 64%, indicating good proficiency in the skills assessed. Benchmarking against the CTCS-SP, these scores therefore indicate a level of competency well above a 50% minimum standard. It should be noted that this assessment was completed following Phase 2 of the training, before the therapists’ pilot cases; this means they had further opportunities to practice and consolidate their skills in Phase 3.

### Patient Adherence

All patients showed excellent adherence. Each patient completed all of the core treatment modules. Overall, patients were issued with a mean of 13.7 modules (SD 1.4) and completed a mean of 12.8 modules (SD 1.7). They spent an average of 40.2 hours (SD 26.4) using the website in the 14-week treatment period. As the program includes an automatic logout feature following periods of inactivity, the activity recorded on the site is thought to be a fair reflection of the time spent actively using the program.

### Internet-Based Cognitive Therapy for Social Anxiety Disorder Patient Outcomes

Mean scores on all outcome measures are shown in Table 2 and effect sizes in Table 3. On the primary patient outcome measure, the LSAS, the mean decrease across the weekly treatment period was 53.8 points (SD 39.5). The within-group effect size (Cohen *d*) was 2.06 (95% CI 0.66-3.46), indicating an effect of medium to very large magnitude in the direction of reduced social anxiety. These figures are at least as good as data from the developers of the treatment [22], where the mean pre-post LSAS decrease was 40.2 points and the effect size was 1.64. Effect size point estimates for the present secondary outcome measures were also large, ranging from 0.92 to 1.89, with the lower limit of the CIs falling in the small-to-medium range for most, though not all, measures. Although highly tentative because of the sample size, these findings suggest the treatment was effective in reducing social anxiety, negative social cognitions, safety behaviors, worry, and functional impairment, and increasing social participation, but did not show a reliable effect on depressed mood or social satisfaction.

**Table 2:** Means and SDs at pretreatment, posttreatment, and 3-month follow-up (N=6).

Measure	Pretreatment, mean (SD)	Posttreatment, mean (SD)	3-Month follow-up, mean (SD)
LSAS^a^	75.8 (30.4)	22.0 (21.0)	17.8 (17.2)
SPWSS^b^	31.7 (11.0)	14.0 (8.1)	14.0 (8.9)
SPIN^c^	43.3 (16.5)	20.0 (12.2)	18.7 (16.9)
FNE^d^	25.2 (3.0)	19.3 (8.5)	18.7 (9.8)
SPS^e^	32.7 (20.4)	12.7 (7.4)	9.7 (9.4)
SIAS^f^	53.3 (16.2)	28.7 (10.1)	27.3 (12.2)
SCQ-f^g^	3.1 (1.1)	1.5 (0.6)	1.5 (0.8)
SCQ-b^h^	47.7 (20.5)	15.6 (13.5)	14.5 (20.3)
SBQ^i^	43.0 (13.2)	22.7 (9.5)	27.7 (17.0)
SAQ^j^	214.5 (44.1)	146.3 (39.2)	159.7 (73.8)
Social participation	37.2 (5.8)	53.5 (14.9)	60.3 (18.5)
Social satisfaction	17.5 (5.8)	22.8 (3.2)	22.5 (8.4)
PHQ^k^	11.3 (7.1)	5.2 (2.6)	6.2 (5.7)
GAD^l^	13.2 (4.5)	6.3 (3.3)	6.8 (5.9)
WSAS^m^	19.5 (7.8)	8.5 (4.2)	9.2 (9.2)

^a^LSAS: Liebowitz Social Anxiety Scale.

^b^SPWSS: Social Phobia Weekly Summary Scale.

^c^SPIN: Social Phobia Inventory.

^d^FNE: Fear of Negative Evaluation Scale.

^e^SPS: Social Phobia Scale.

^f^SIAS: Social Interaction Anxiety Scale.

^g^SCQ-f: Social Cognitions Questionnaire (frequency; mean score).

^h^SCQ-b: Social Cognitions Questionnaire (belief; mean score).

^i^SBQ: Social Behavior Questionnaire.

^j^SAQ: Social Attitudes Questionnaire.

^k^PHQ: Patient Health Questionnaire.

^l^GAD: Generalized Anxiety Disorder Questionnaire.

^m^WSAS: Work and Social Adjustment Scale.

**Table 3:** Within-group effect sizes between pretreatment, posttreatment, and 3-month follow-up (N=6).

Measure	Cohen *d* (95% CI)		
	Pre-to-Post	Pre-to-Follow-up	Post-to-Follow-up
LSAS^a^	2.06 (0.66 to 3.46)	2.34 (0.88 to 3.81)	0.22 (−0.92 to 1.35)
SPWSS^b^	1.83 (0.48 to 3.18)	1.76 (0.43 to 3.10)	0.00 (−1.13 to 1.13)
SPIN^c^	1.61 (0.31 to 2.91)	1.48 (0.20 to 2.75)	0.09 (−1.04 to 1.22)
FNE^d^	0.92 (−0.27 to 2.10)	0.90 (−0.29 to 2.09)	0.07 (−1.06 to 1.20)
SPS^e^	1.30 (0.06 to 2.55)	1.45 (0.18 to 2.72)	0.35 (−0.79 to 1.49)
SIAS^f^	1.82 (0.48 to 3.17)	1.81 (0.46 to 3.15)	0.12 (−1.01 to 1.25)
SCQ-f^g^	1.89 (0.53 to 3.25)	1.71 (0.39 to 3.03)	−0.02 (−1.15 to 1.11)
SCQ-b^h^	1.85 (0.50 to 3.20)	1.63 (0.32 to 2.93)	0.06 (−1.07 to 1.19)
SBQ^i^	1.76 (0.43 to 3.10)	1.01 (−0.19 to 2.21)	−0.36 (−1.50 to 0.78)
SAQ^j^	1.63 (0.33 to 2.94)	0.90 (−0.29 to 2.09)	−0.23 (−1.36 to 0.91)
Social participation	1.45 (0.17 to 2.72)	1.69 (0.37 to 3.01)	0.41 (−0.74 to 1.55)
Social satisfaction	1.14 (−0.08 to 2.36)	0.69 (−0.47 to 1.86)	−0.05 (−1.18 to 1.08)
PHQ^k^	1.15 (−0.07 to 2.37)	0.80 (−0.38 to 1.98)	−0.22 (−1.36 to 0.91)
GAD^l^	1.72 (0.39 to 3.04)	1.20 (−0.03 to 2.43)	−0.10 (−1.24 to 1.03)
WSAS^m^	1.75 (0.42 to 3.08)	1.21 (−0.02 to 2.44)	−0.09 (−1.23 to 1.04)

^a^LSAS: Liebowitz Social Anxiety Scale.

^b^SPWSS: Social Phobia Weekly Summary Scale.

^c^SPIN: Social Phobia Inventory.

^d^FNE: Fear of Negative Evaluation Scale.

^e^SPS: Social Phobia Scale.

^f^SIAS: Social Interaction Anxiety Scale.

^g^SCQ-f: Social Cognitions Questionnaire (frequency; mean score).

^h^SCQ-b: Social Cognitions Questionnaire (belief; mean score).

^i^SBQ: Social Behavior Questionnaire.

^j^SAQ: Social Attitudes Questionnaire.

^k^PHQ: Patient Health Questionnaire.

^l^GAD: Generalized Anxiety Disorder Questionnaire.

^m^WSAS: Work and Social Adjustment Scale.

Changes in scores from posttreatment to 3-month follow-up were analyzed to review the maintenance of treatment gains over this period. Patients retained access to the program during this 3-month phase, though therapist contact was reduced to just one brief phone call at monthly intervals. As shown in Table 3, the effect size point estimates for most measures ranged between −0.20 and 0.20, meaning the threshold for a small effect in either direction was not reached. This suggested that the improvements from treatment had been maintained over this period. There was some evidence of a small effect in the direction of further improvement on the LSAS, SPS, and social participation measures during this period. On the SBQ, SAQ, and PHQ, there was some evidence of a small effect indicating a deterioration. Although this may indicate a lessening of some secondary treatment effects during the booster phase, these results may have been heavily influenced by the scores of 1 patient who did not respond to treatment and showed a decline on secondary outcome measures over the booster phase. Again, the CIs for these effect sizes were extremely wide because of sample size, so they can only provide a preliminary indication of the maintenance of treatment gains.

**Figure 2:**
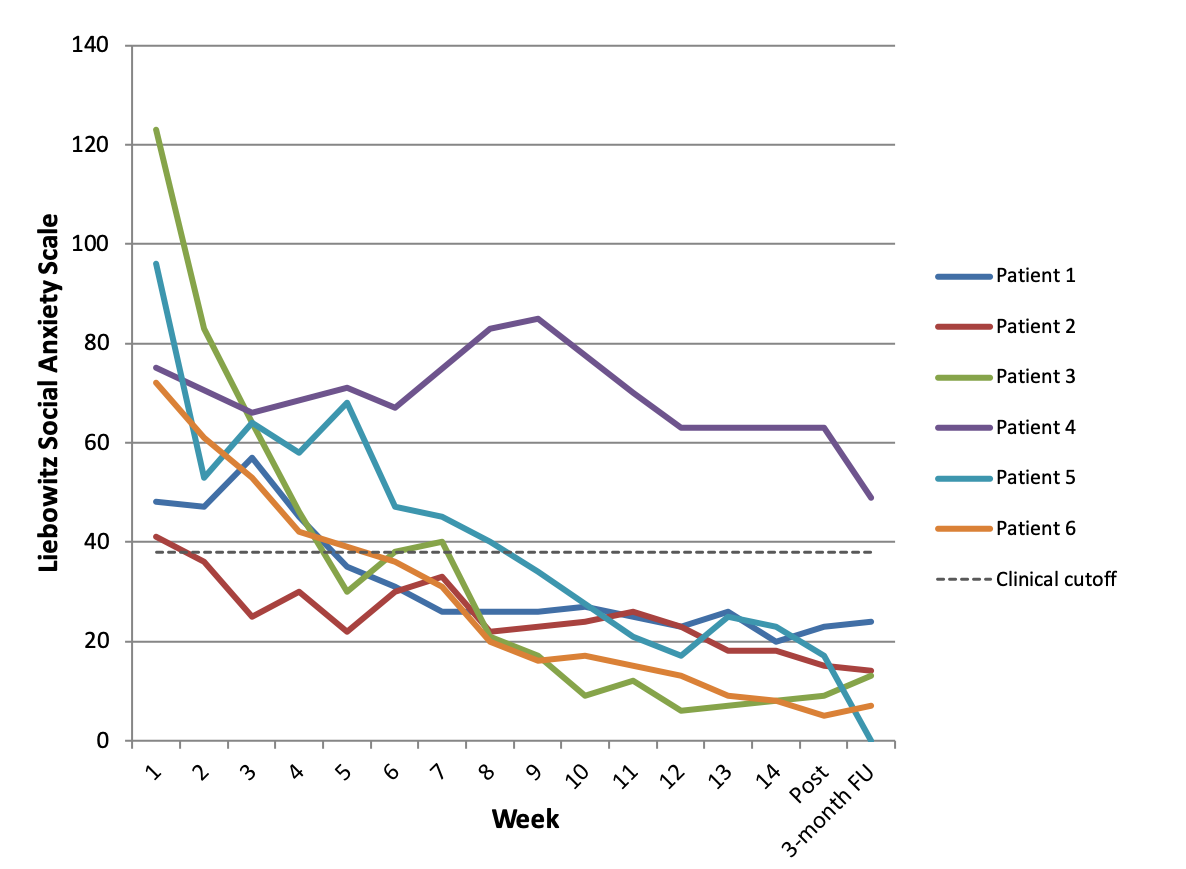
Weekly scores on the Liebowitz Social Anxiety Scale (self-report) across treatment for the 6 pilot patients. Week 15 represents the posttreatment assessment. The dotted line represents the clinical cutoff score of 38 points.

Overall, 5 of the 6 patients showed an improvement on the LSAS greater than 31% at the posttreatment assessment and were thus classified as treatment responders. All 5 patients were also classified as remitted from their social anxiety, showing a drop of at least 12 points and falling below the clinical cutoff at posttreatment. Individual LSAS scores over time are shown in Figure 2. Using the IAPT criteria based on social anxiety (SPIN) and depression (PHQ) scores, 4 patients showed reliable improvement and 3 were classified as reliably recovered from SAD.

The 1 patient who was not classified as a responder to treatment within the weekly treatment phase experienced difficulties with depressed mood, which may have impacted their motivation to log in and work on the treatment website and to complete behavioral experiments. When compared with some other patients, they spent relatively little time on the website (12.5 hours total) and completed fewer behavioral experiments. Across the patient cohort, the amount of time spent on the website was positively correlated with percentage change on the LSAS (*r*=.584). It is also noted that the 2 patients with relatively lower baseline LSAS scores, because of more situation-specific forms of social anxiety, finished treatment with scores higher than some other patients whose baseline scores were more severe. The correlation between baseline LSAS score and percentage change on the LSAS was *r*=.443.

### Patient and Therapist Feedback

All 6 patients completed the anonymous feedback survey at the end of the booster period. Given the small sample size, the frequencies of responses are reported. Overall, 5 of the patients felt that the program was relatively easy to understand in English, though 1 experienced some difficulties expressing their ideas and feelings and felt this could be a barrier for the therapy (respondent 4). Patients reported various therapist behaviors that they found helpful, including sending a written summary following phone calls, helping to generate new perspectives, identifying details of modules they may have missed, advising on behavioral experiments to try, and helping them to review their week. In terms of the treatment as a whole, respondents reported liking the phone calls (described as “intensive but helpful” by respondent 1); the practical nature of the contents; the effort, help, and professionalism of the therapist; the inclusion of both general and specific content; and the way the questionnaires allowed them to review their emotional state. In addition, 4 patients did not report any dislikes about the treatment. For security reasons, iCT-SAD uses 2-factor authentication at log-in, and 1 participant disliked having to enter an authentication code each time (respondent 1). One participant felt it was sometimes hard to motivate themselves to finish the tasks and expressed a concern that they may not have understood their own anxiety very well (respondent 4). All patients rated the program as easy to use on a desktop or laptop computer, but 2 reported difficulty when using a tablet or mobile phone. In sum, responses indicated a good level of treatment satisfaction and acceptability, with some but relatively few technical or practical difficulties.

Therapists’ feedback indicated that they did not think any major cultural adaptation was required to the program content or implementation procedures for the Hong Kong context. However, the therapists did identify certain aspects of the iCT-SAD program that may prove difficult to understand when patients are working in a nonnative language. This included some video and audio material where groups of people are chatting informally, perhaps talking at the same time or in incomplete sentences. In addition, one of the exercises used in the program to practice externally focused attention asks patients to watch a video of someone reading a story to them while they alternate their attention between focusing on themselves and on the story content.

## Discussion

### Principal Findings

This study has provided an example of a procedure for training therapists to deliver a guided internet intervention, including details of the content covered, and how progress was evaluated. The results indicated that the training was effective in improving therapists’ knowledge and skills in relation to iCT-SAD. The 3 therapists achieved a good level of competence when objectively measured using the skills test following Phase 2 of training. They were then able to consolidate these skills in their work with pilot cases, as demonstrated by the positive outcomes achieved by this cohort. These results suggested that the clinical outcomes for patients in Hong Kong undertaking iCT-SAD with local therapists were comparable to those seen in a UK study from the developers of the treatment [22].

The 6 patients showed good adherence to the iCT-SAD program, completing treatment modules, phone calls with the therapist, behavioral experiments, and weekly questionnaire measures. All patients completed the full weekly and booster phases of treatment, indicating a good level of treatment acceptability and feasibility in this setting and sufficiently reliable functioning of the website. The therapists successfully implemented the treatment in line with the protocol and no significant deviations from this were required. These findings are of course preliminary given the present sample size and an RCT would be required to establish the feasibility and efficacy of the program in Hong Kong more robustly.

This study took the approach of piloting the intervention in its unadapted form, meaning that the results could be benchmarked against the UK data and feedback could be elicited from both patients and therapists. Promisingly, the mean pre-post change on the LSAS and associated effect size point estimate (Cohen *d*=2.06) were large, suggesting that the high level of efficacy observed in the United Kingdom appears to have been maintained within a different cultural setting. Patient and therapist feedback did not identify any major cultural or linguistic barriers; the suggestions made to assist understanding were modest. We have since created some additional Chinese resources for the components of the program mentioned along with English-subtitled versions of the site videos. Overall, the findings in this study suggest that the approach of starting with the unadapted intervention was justified given the clinical outcomes obtained and that the adaptations suggested were quite modest and smaller than might have been expected at the outset of the project.

### Limitations

Limitations of this study include the small number of therapists trained and the fact that the self-report assessment and skills test were developed specifically for this study and are therefore not yet externally validated. As mentioned above, the small number of patients in this pilot study can only tentatively indicate treatment efficacy and linguistic or cultural considerations, and larger controlled studies are therefore required. The training program implemented here was intensive and was not designed to be scalable at this stage. Future work should consider the best methods to provide such training in less time and to larger groups of therapists. In developing training programs for internet interventions that are based closely on a face-to-face treatment, our view is that familiarity with the face-to-face protocol is an important preliminary step and that treating a small number of training cases online under clinical supervision is essential for applying and consolidating skills. Online therapist training methods are likely to be the most scalable approach for the future, but preliminary work such as the approach taken here can help to develop a clearer understanding of what training should cover and how. The aim should be to determine what therapists need from training, making it easier to design online materials for that purpose. Our research team has recently started to develop online therapist training materials for iCT-SAD, which are available on our resources website [30]. Further research could also usefully explore whether less-experienced therapists can be trained to achieve similar outcomes.

**Conclusions**

This study has described and evaluated a program of therapist training in iCT-SAD, which appeared effective at increasing therapists’ knowledge and skills and resulted in positive clinical outcomes among a pilot case series of 6 patients in Hong Kong, with initial evidence that these were sustained at 3-month follow-up. The treatment appeared feasible and acceptable in this setting. An RCT of the iCT-SAD treatment in Hong Kong is now in progress. Given that therapist training is critical to the successful dissemination of internet interventions, further research to develop, describe, and evaluate therapist training procedures is recommended.

## Appendix

Multimedia Appendix 1 Internet-based Cognitive Therapy for social anxiety disorder self-evaluation assessment.

Multimedia Appendix 2 Internet-based Cognitive Therapy for social anxiety disorder skills test.

## Abbreviations

CT: Cognitive Therapy
CTCS-SP: Cognitive Therapy Competence Scale for Social Phobia
CT-SAD: Cognitive Therapy for social anxiety disorder
GAD: Generalized Anxiety Disorder
IAPT: Improving Access to Psychological Therapies
iCT: internet-based Cognitive Therapy
iCT-SAD: internet-based Cognitive Therapy for social anxiety disorder
LSAS: Liebowitz Social Anxiety Scale
PHQ: Patient Health Questionnaire
RCT: randomized controlled trial
SAD: social anxiety disorder
SAQ: Social Attitudes Questionnaire
SBQ: Social Behavior Questionnaire
SPIN: Social Phobia Inventory
SPS: Social Phobia Scale
