# Supplementary material for: Internet-Based Cognitive Therapy for Social Anxiety Disorder in Hong Kong: Therapist Training and Dissemination Case Series
Source: JMIR Form Res. 2019 May 15;3(2):e13446. doi: 10.2196/13446 (PMC6540721; doi:10.2196/13446)
Supplement: Multimedia Appendix 1 [file formative_v3i2e13446_app1.docx]

**Self-Evaluation for Internet-based Cognitive Therapy**

This scale is designed to help therapists consider their understanding and implementation of a range of techniques used in online cognitive therapy treatments. It aims to help therapists reflect on their knowledge and skills in using these programmes, and to help supervisors and trainers identify the best ways to help therapists develop further.

Please answer the following:

| Name: |  | |
| --- | --- | --- |
| Job Title: |  | |
| CBT qualification(s): | None  Diploma  Masters  DClinPsy or equivalent  Other (please specify) | ☐  ☐  ☐  ☐  ☐ |
| Number of Years in Qualified Practice: |  | |
| Number of years practising CBT: |  | |
| Number of clients treated using guided online CBT treatments: |  | |
| Number of clients treated using iCT-SAD: |  | |

**For each of the therapy skills listed below, please provide two ratings:**

**Knowledge:** Currently, how much do you know about the skill or technique?

| **0** | **1** | **2** | **3** | **4** | **5** | **6** | **7** | **8** |
| --- | --- | --- | --- | --- | --- | --- | --- | --- |
| No knowledge |  | Limited knowledge |  | Some knowledge |  | Good knowledge |  | Extensive knowledge |

**Skills:** Currently, how well do you think you can apply the skill or technique?

| **0** | **1** | **2** | **3** | **4** | **5** | **6** | **7** | **8** |
| --- | --- | --- | --- | --- | --- | --- | --- | --- |
| No skills |  | Limited skills |  | Some skills |  | Good skills |  | Extensive skills |

**Part 1: General Online Skills**

|  | **Knowledge**  **(0-8)** | **Skills**  **(0-8)** |
| --- | --- | --- |
| Developing and maintaining client engagement with the online programme |  |  |
| Developing and maintaining a supportive online therapeutic relationship with the client |  |  |
| Reviewing clients’ work on the site in detail |  |  |
| Identifying and addressing potential barriers to progress using the online programme |  |  |
| Supporting clients to extract the key messages and learning from therapy activities |  |  |
| Contacting clients with appropriate frequency and regularity |  |  |
| Writing messages that effectively support and guide clients through the programme |  |  |
| Managing situations where clients are not completing work on the site, and/or not responding to messages |  |  |
| Maintaining clear and detailed therapy notes for online clients |  |  |
| Using supervision effectively in relation to online clients |  |  |
| Helping clients navigate and use the site content and technical features |  |  |

**Part 2: iCT-SAD Specific Skills**

| **Phone calls** | **Knowledge**  **(0-8)** | **Skills**  **(0-8)** |
| --- | --- | --- |
| Scheduling phone calls appropriately based on the treatment protocol |  |  |
| Setting an appropriate agenda for each call, prioritising the most important content |  |  |
| Making reference back to appropriate module content |  |  |
| Agreeing appropriate homework/action points, with associated timescales |  |  |
| Keeping phone calls to their allotted duration (15-20 minutes) |  |  |
| Following a call, using appropriate strategies to summarise and remind clients about agreed homework |  |  |

| **Therapy Components** | **Knowledge**  **(0-8)** | **Skills**  **(0-8)** |
| --- | --- | --- |
| Identifying and clarifying clients’ goals online, including their timescale in relation to treatment |  |  |
| Supporting clients to formulate an appropriate and detailed individualised cognitive model online, and using this as a guide throughout treatment |  |  |
| Conducting the ‘self-focused attention and safety behaviours experiment’ via webcam |  |  |
| Suggesting a third condition in the self-focused attention and safety behaviours experiment when appropriate |  |  |
| Supporting clients to undertake videofeedback online |  |  |
| Suggesting strategies to overcome problems clients may experience with videofeedback |  |  |
| Supporting clients with attention training online, including ongoing practice |  |  |
| Helping clients to plan and schedule behavioural experiments relevant to their concerns |  |  |
| Supporting clients in implementing behavioural experiments, including those that are more challenging (e.g. decatastrophising experiments) |  |  |
| Helping clients to extract maximal learning from behavioural experiments, and making suggestions to develop experiments further |  |  |
| Selecting optional treatment modules in order to individualise the treatment plan |  |  |
| Supporting clients to use the virtual audiences feature of the site to record and view presentations |  |  |
| Supporting clients to use discrimination training and imagery rescripting to address problematic images and memories |  |  |
| Reviewing the client’s blueprint constructively |  |  |
| Preparing a detailed plan with clients for the follow-up period |  |  |
| Understanding the methods to monitor clients’ activity using the online programme and maintaining a detailed understanding of their clinical progress |  |  |
| Understanding and implementing the iCT-SAD programme according to the treatment protocol |  |  |
