# Supplementary material for: Internet-Based Cognitive Therapy for Social Anxiety Disorder in Hong Kong: Therapist Training and Dissemination Case Series
Source: JMIR Form Res. 2019 May 15;3(2):e13446. doi: 10.2196/13446 (PMC6540721; doi:10.2196/13446)
Supplement: Multimedia Appendix 2 [file formative_v3i2e13446_app2.docx]

**iCT-SAD Skills Test**

Please read the below information and imagine you are working with the clients described using the iCT-SAD programme.

**Client 1: Anna, 37**

Anna has just started treatment. Some of her assessment questionnaires are shown below:

**Liebowitz Social Anxiety Scale (LSAS)**


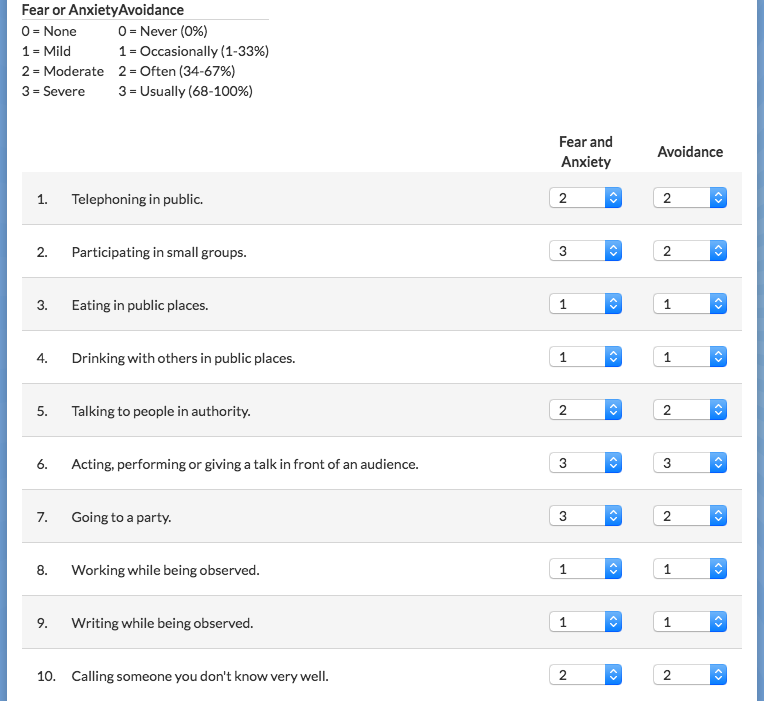


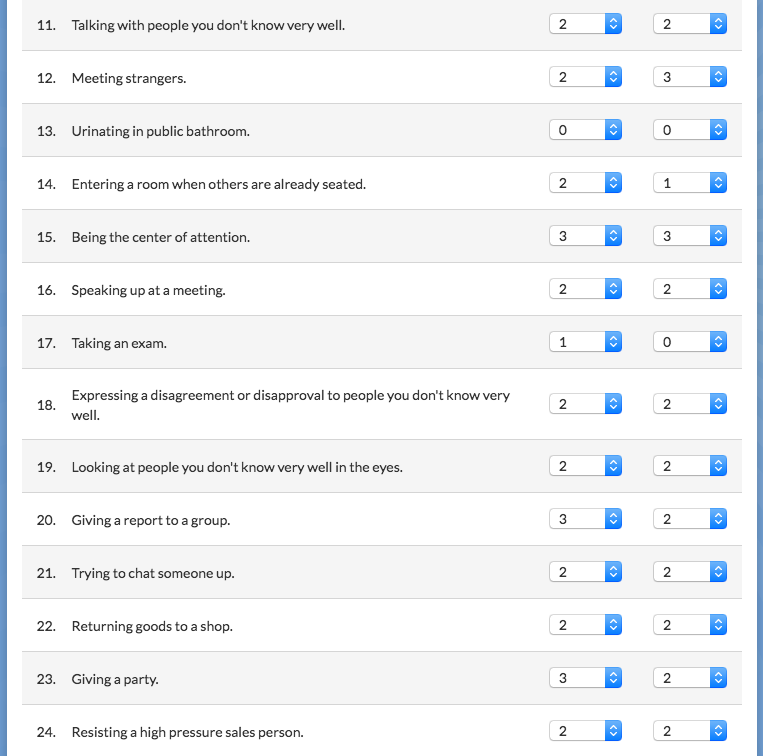


**Social Cognitions Questionnaire (SCQ)**


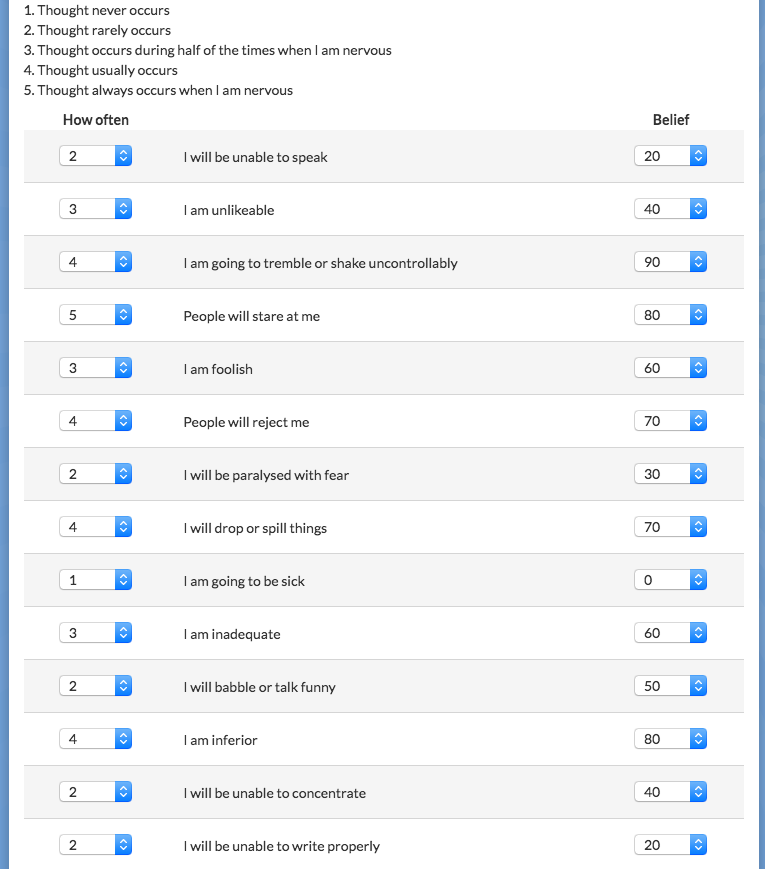


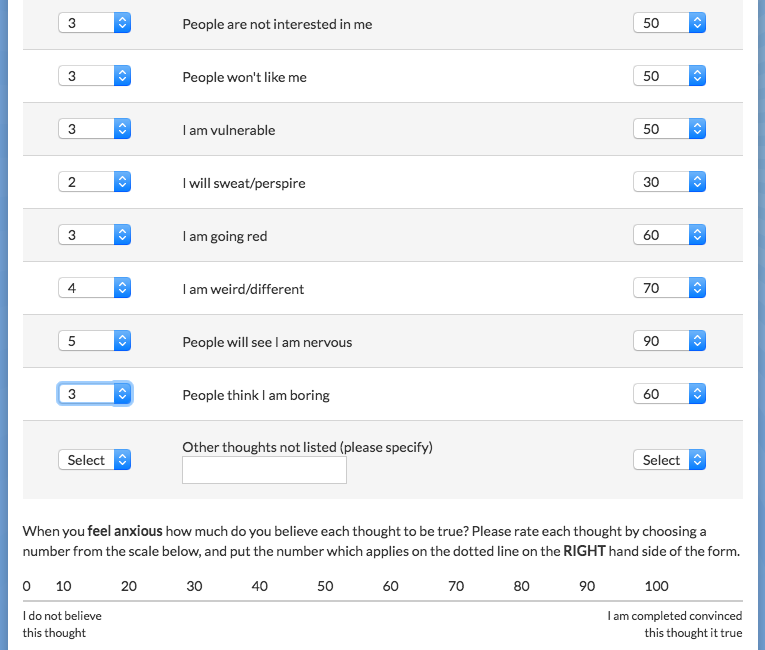


**Social Summary Rating Scale**

**
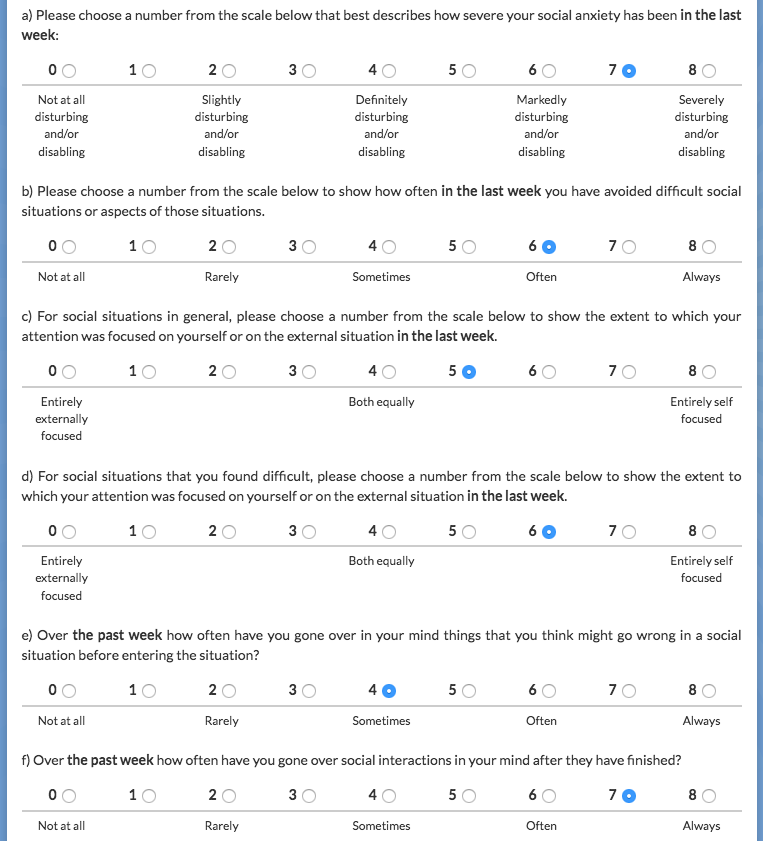
**

**Social Behaviours Questionnaire (SBQ)**


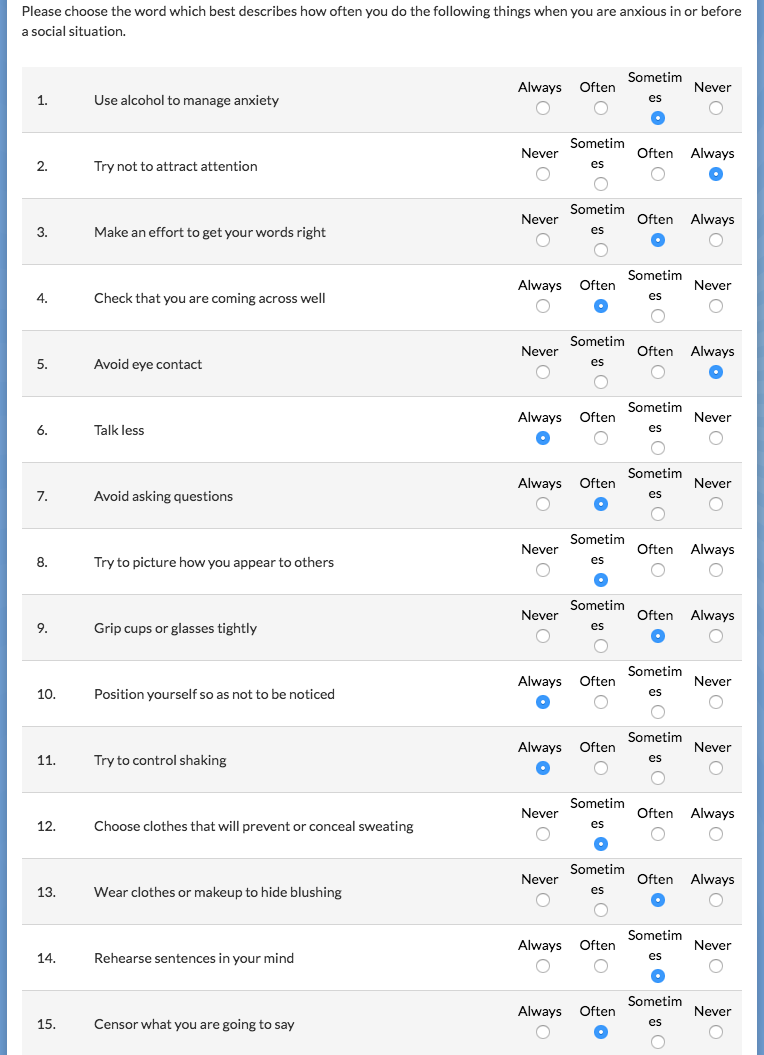


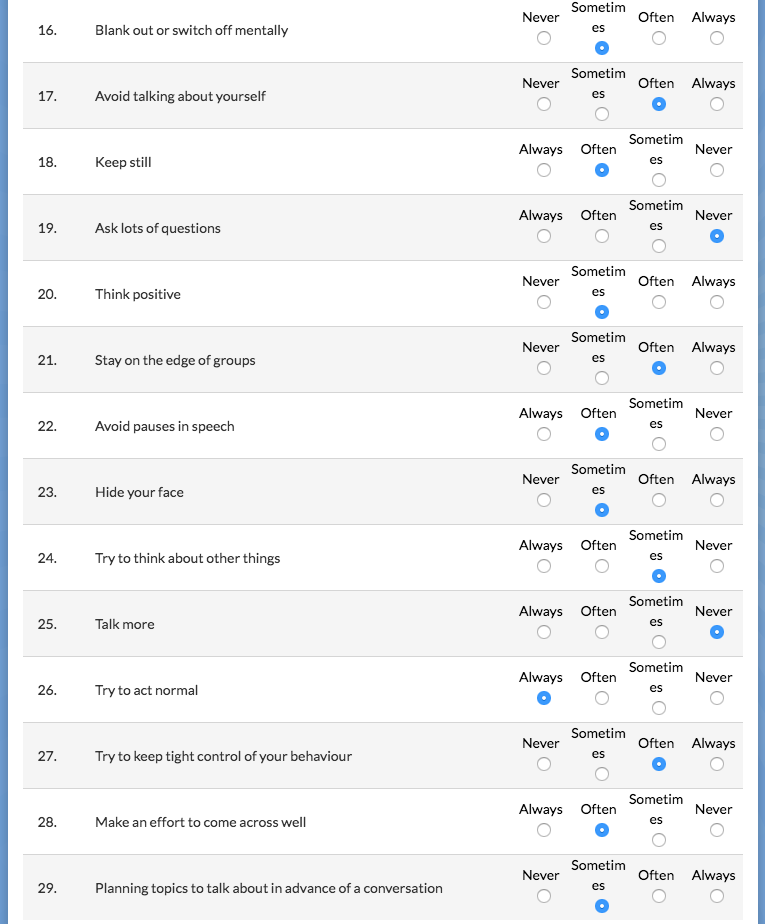


**Patient Health Questionnaire (PHQ9)**


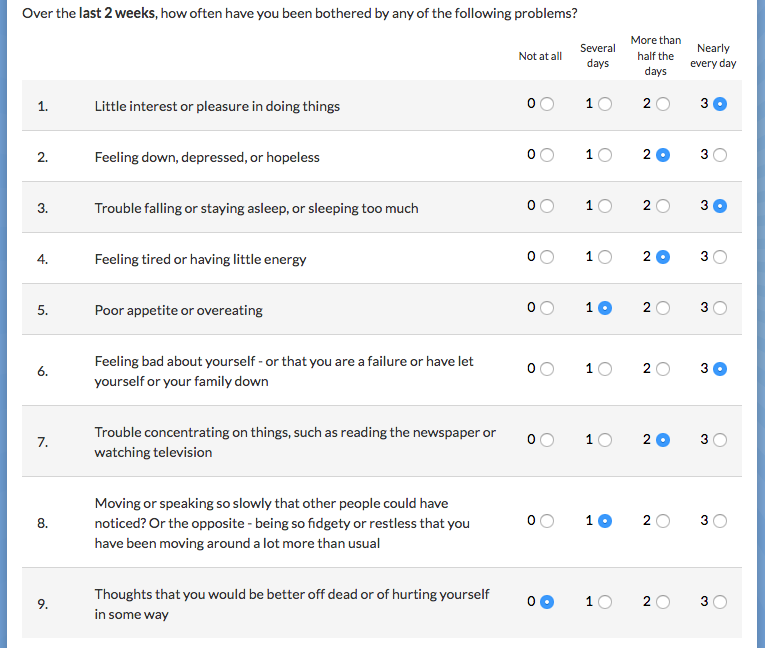


*******************************************************************************

**Looking through these questionnaires, which of the optional treatment modules would be most helpful for Anna across her treatment? Please say why you have chosen each one.**

The model that Anna has completed in the ‘Getting Started’ module is shown below:


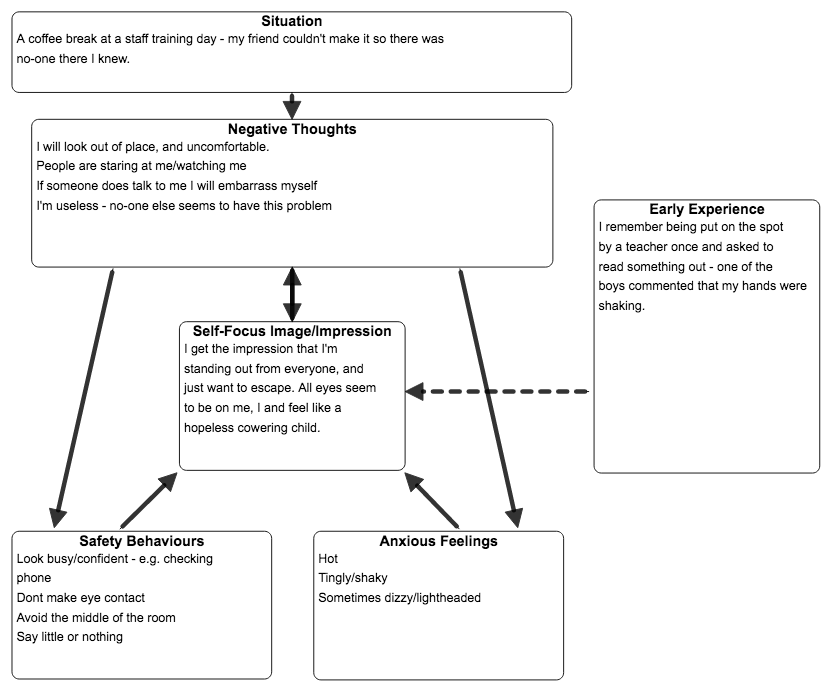


*******************************************************************************

**Please write brief notes on what you would want to discuss about the model with Anna in your next call. Is there anything you would want further information on, or that seems to be missing?**

**Client 2: Olivia, 33**

Olivia is in week 2 of treatment, and you are completing the ‘My Attention and Safety Behaviour Experiment’ module with her via webcam. The comparison of her scores for conversations 1 and 2 is shown below:


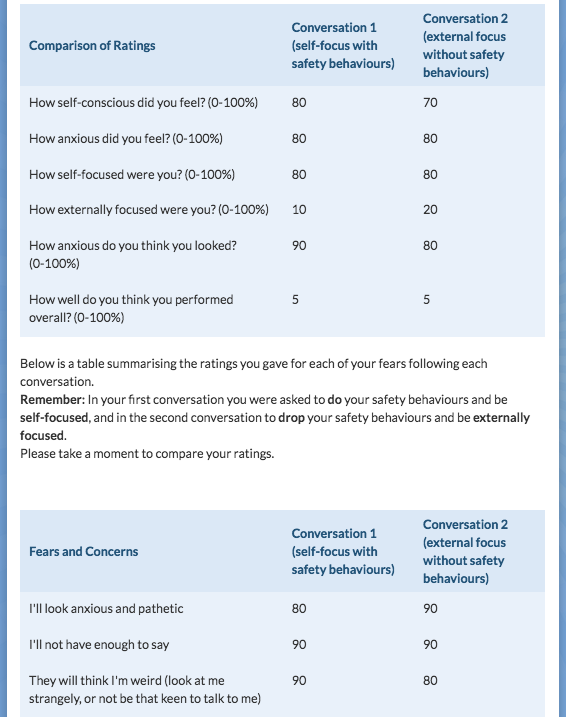


*******************************************************************************

**What appears to be the problem here, and how would you continue the webchat with Olivia at this point?**

**Client 3: Marcus, 24**

Marcus is in week 3 of treatment, and you have a phonecall scheduled this afternoon. He was due to be completing the ‘Behavioural Experiments’ and ‘Getting out of your head and into the world’ modules. However, you can see from the site that he has not logged in during the past week, and although you have sent messages and SMS reminders you have not heard from him since your previous call.

*******************************************************************************

**Please write an agenda for today’s phonecall below, with brief notes about what you would want to discuss:**

**Client 4: Anisha, 29**


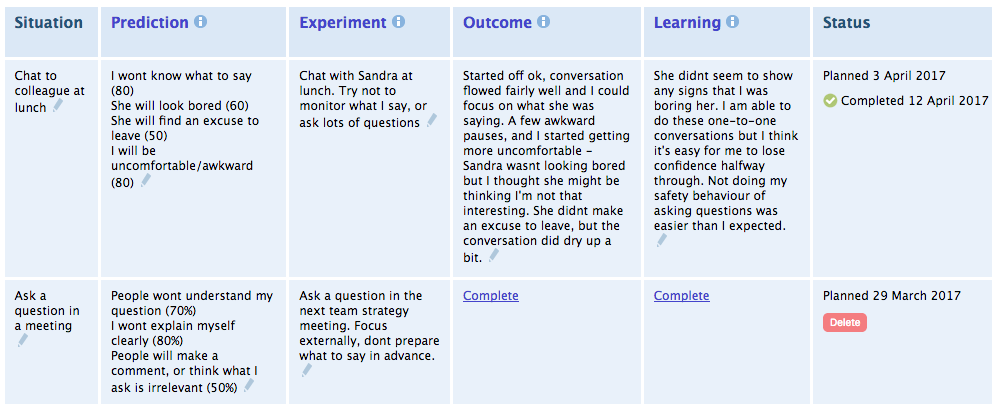
Anisha is in week 5 of treatment, and you have been helping her to plan and implement some behavioural experiments. Below are the most recent experiments from her behavioural experiment log:

*******************************************************************************

**How would you respond to Anisha? Please write brief notes on the points you would raise with her:**

**Client 5: Connor, 19**

Connor has recently completed the ‘Feeling Boring’ module. His responses to part of this module are shown below:


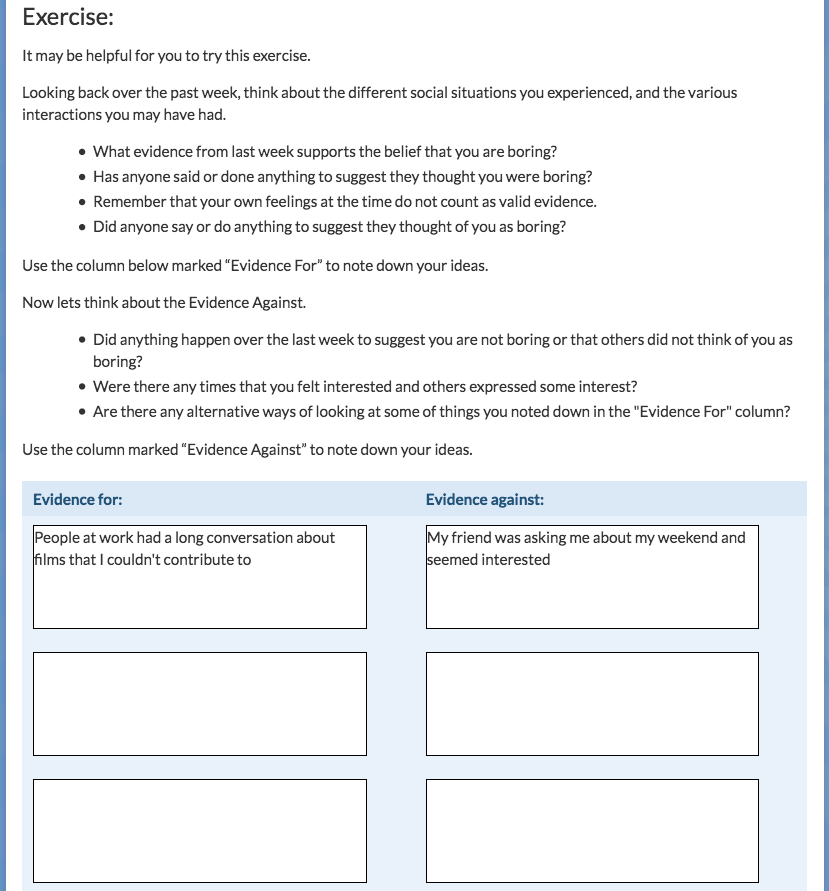


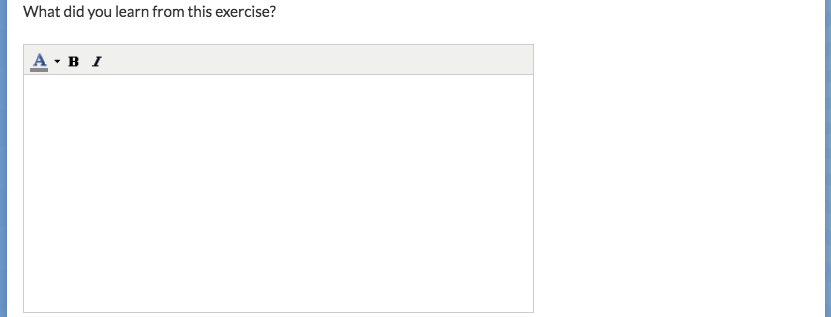


*******************************************************************************

**What would you want to ask or discuss with Connor to help him with this exercise? What would you be hoping to achieve?**

**Client 6: Julie, 54**

Julie is in week 10 of treatment. She has made steady progress and is currently working on the ‘Leaving the Past Behind’ module. She has sent you the following message:

*Thanks for your message, sorry for the delay in getting back to you.*

*I’ve been finding things quite hard going recently – some difficulties with family at the moment, and busy at work, so by the time I get home I’m exhausted and don’t really feel motivated or focused enough to work on the site. I don’t want to let you down, and I have been finding things helpful so far, but it’s just a struggle at the moment…*

*Julie*

*******************************************************************************

**Please write a message in response to Julie below:**

**Client 7: Simon, 40**

Simon is in week 14 of treatment and is about to move into the follow-up phase. He has just messaged you to say he has completed the blueprint module. An extract is shown below:


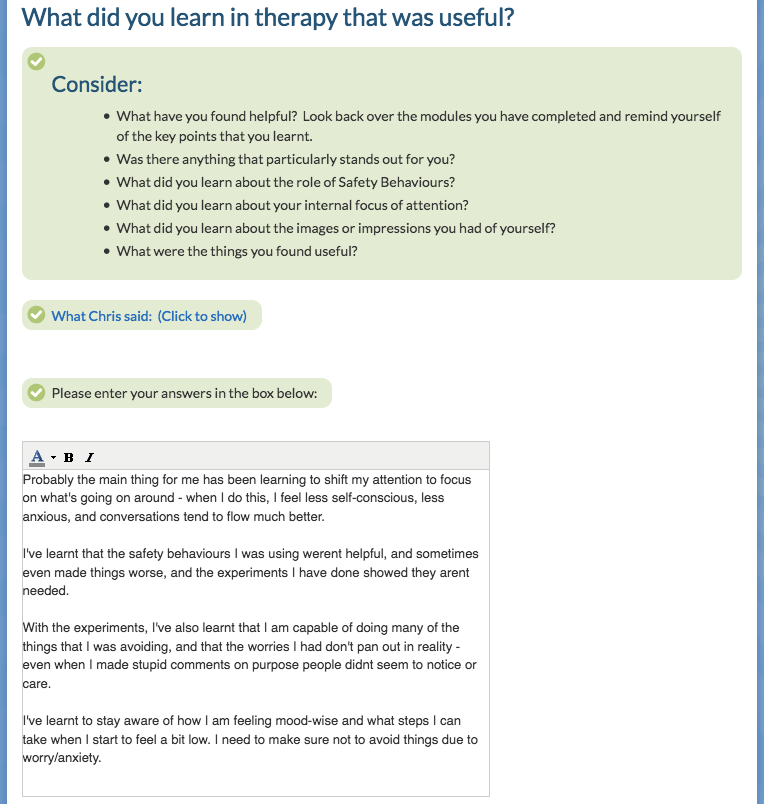


*******************************************************************************

**What suggestions might you make to Simon to help him improve this response?**
